# Horse odor exploration behavior is influenced by pregnancy and age

**DOI:** 10.3389/fnbeh.2022.941517

**Published:** 2022-07-28

**Authors:** Maria Vilain Rørvang, Klára Nicova, Jenny Yngvesson

**Affiliations:** ^1^Department Biosystems and Technology, Swedish University of Agricultural Sciences, Lomma, Sweden; ^2^Department of Ethology, Institute of Animal Science, Prague, Czechia

**Keywords:** olfaction, trigeminal nerve, horse-human relationship, smell, sensory ability, scent, enrichment, nose-work

## Abstract

In spite of a highly developed olfactory apparatus of horses, implying a high adaptive value, research on equine olfaction is sparse. Our limited knowledge on equine olfaction poses a risk that horse behavior does not match human expectations, as horses might react fearful when exposed to certain odors, which humans do not consider as frightening. The benefit of acquiring more knowledge of equine olfaction is therefore twofold; (1) it can aid the understanding of horse behavior and hence reduce the risk of dangerous situations, and (2) there may be unexplored potential of using odors in several practical situations where humans interact with horses. This study investigated behavior and olfactory sensitivity of 35 Icelandic horses who were presented with four odors: peppermint, orange, lavender and cedar wood in a Habituation/Dishabituation paradigm. The response variables were sniffing duration per presentation and behavioral reaction (licking, biting, snorting, and backing), and data were analyzed for potential effects of age, sex and pregnancy. Results showed that habituation occurred between successive odor presentations (1st vs. 2nd and 2nd vs. 3rd presentations: *P* < 0.001), and dishabituation occurred when a new odor was presented (1st vs. 3rd presentations: *P* < 0.001). Horses were thus able to detect and distinguish between all four odors, but expressed significantly longer sniffing duration when exposed to peppermint (peppermint vs. orange, lavender and cedar wood: *P* < 0.001). More horses expressed licking when presented to peppermint compared to cedar wood and lavender (*P* = 0.0068). Pregnant mares sniffed odors less than non-pregnant mares (*P* = 0.030), young horses (age 0-5 years) sniffed cedar wood for longer than old horses (*P* = 0.030), whereas sex had no effect (*P* > 0.050). The results show that horses’ odor exploration behavior and interest in odors varies with age and pregnancy and that horses naïve to the taste of a substrate, may be able to link smell with taste, which has not been described before. These results can aid our understanding of horses’ behavioral reactions to odors, and in the future, it may be possible to relate these to the physiology and health of horses.

## Introduction

Odors are a constant component of an animal’s environment, and play a key role in the expression of behavior, both as a stimulus and as a modulator of the behavior elicited by another stimulus. In spite of this, the role of olfaction is often ignored in animal behavior studies, and when handling and interacting with horses. As a result, most of the currently existing knowledge about olfaction originate from human research or rodents constituting human models (Nielsen et al., 2015). The lack of knowledge from animals poses a risk that the role of olfaction is underestimated in animal studies and animal handling.

In domestic horses, olfaction is important in the development and expression of behavior (odors can both function as stimuli leading to behavior, and as modulators of the behavioral response following another stimulus), in many contexts of horses’ lives. Food flavor, foraging, and social behavior (Hothersall et al., 2010; Jezierski et al., 2018), mating and reproduction (Marinier et al., 1988; Briant et al., 2010; Jezierski et al., 2018) but also in the evaluation of predation risk (Nielsen et al., 2015) to name but a few. It is therefore surprising how sparse research on the olfactory abilities of horses is (Rørvang et al., 2020). As a result, this study builds on the literature available on equids but when knowledge on equids is lacking, we relied on research from rodents or other mammals.

### The role of olfaction in equitation

Horses are sensitive to and affected by odors in their surroundings. These abilities are mentioned in equitation science books (McGreevy, 2004; McGreevy et al., 2018), but are also often ignored in practice. How horses respond to odors is important (1) as it plays a key role in their everyday life (Nielsen, 2017), and hence their welfare, and (2) as horse’s reactions, and the ability to predict these reactions, are crucial for humans to ensure safety when handling and training horses (Rørvang et al., 2020). An odor may be neutral to the horse, but it might also elicit either avoidance behavior or have an attractive effect depending on the horse’s perception of the odor. Studies have shown that avoidance and vigilance behavior was shown by horses when a predator odor (wolf urine) or an unknown odor (eucalyptus oil) was present, but in addition, this was not associated with higher heart rates (Christensen et al., 2005). The latter might indicate that although horses expressed behavioral signs of fear, the body’s physiological response was not responding to a perceived danger.

It is not uncommon practice for various odorants of non-social origin (i.e., odors not derived from horses such as odors contained within excrements) to be used for calming horses in stressful situations (e.g., during trailering, Heitman et al., 2018) and as aromatherapy for horses (e.g., Kosiara et al., 2021). Moreover, a few years ago (2020, Draaisma, 2021), a new trend in horse training appeared. The so-called nose work exercise, which has been developed as a leisurely activity with horses. Nose- or scent-work for horses consists of placing an odor in/at a designated place in the horse’s environment either indoor or outdoor, and subsequently allowing the horse to sniff out the odor, which then elicits a reward (Draaisma, 2021). Despite the increasing popularity of nose work in horse training, however, there is no scientific background for its applicability or for selecting suitable odors, hence these exercises are mainly based on knowledge from dogs (Duranton and Horowitz, 2019; DeGreeff et al., 2020; Draaisma, 2021). Some trainers thus use knowledge of olfaction in their daily handling and training of horses, without any scientific information of its efficacy and potential effect of individual variation. In addition, use of essential oils such as lavender, lemongrass or peppermint is also gaining more and more practical attention in horse training (Oke, 2021), as these oils are often used either as nose work scents or as presumably, innately calming odors. While studies have confirmed the antimicrobial and antibacterial effects (Mancianti and Ebani, 2020), as well as their efficacy in insecticides (Cox et al., 2020), knowledge on horses ability to detect and differentiate between such odors as well as their preferences of sniffing these odors remain unexplored. More knowledge is thus needed in order to establish if horses can detect such odors and which individual factors affect their odor exploration behavior and interest in the odors.

### The influence of age, sex and gestational stage on olfactory interest

In human research, it is well established that olfactory abilities (like other senses) deteriorate with age (James Evans et al., 1995; Sorokowska et al., 2015). Some diseases can also contribute to dampen the sensitivity, or even change preferences (Doty and Kamath, 2014). Studies of how sensory sensitivity might change with age in non-human mammals are rare, but from studies on working dogs, similar tendencies have been found (Jenkins et al., 2018). Following these results, it may be fair to argue that age could affect olfactory exploration behavior and interest in the horse (Han et al., 2022). On the other hand, one study has assessed if age affected olfactory abilities of horses (Hothersall et al., 2010), and found no effect when mares and foals were exposed to social odors. It is however unknown if age affects horses’ exploration behavior in complex (multiple molecules in contrast to simple odors, i.e., single molecule odors), non-social odors.

Olfaction research in humans shows that females seem to outperform males in their olfactory abilities (Sorokowski et al., 2019). This effect was most pronounced in children, where girls were more aware and reactive toward odorants (Nováková et al., 2017). Findings from other mammalian, non-human species are sparse, but from results in mice studies point to females reacting more rapidly to odor-induced signaling (Kass et al., 2017). From chimpanzees, males and females also differ in their behavior when exposed to odors, with females sniffing more than males during feeding, and vice versa during social interactions (Matsumoto-Oda et al., 2007). Collectively, the findings from these studies can be used to argue for a potential effect of sex on horses’ odor exploration behavior and interest in odors.

Another factor, which might affect olfactory responsiveness and interest, is the gestational stage of a horse. It is well known that pregnant women change in their olfactory preferences during pregnancy (reviewed in Cameron, 2014), but contrarily not much is known about non-human mammalian species on this topic. Research on dairy cows suggest that pregnant dairy cow change with respect to their olfactory responsiveness toward (or perception of) social odors (here: amniotic fluid) as calving approaches (Rørvang et al., 2017a; Jensen and Rørvang, 2018; Rørvang, 2018). These changes are believed to be caused by hormonal changes in the body of the cow as parturition approaches, resulting in the onset of maternal behavior (Levy and Nowak, 2017). It is thus reasonable to propose that pregnant mares might be under the same hormonal influence and as a result differ from non-pregnant mares in olfactory interests.

The olfactory Habituation/Dishabituation test is a simple, and sophisticated method for the assessment of olfactory capacities in animals (Sundberg et al., 1982). The test paradigm relies on the theory; repeated presentation of the same odor resulting in decreased sniffing duration (habituation), whereas subsequent presentation of a new odor reinstates sniffing duration (dishabituation) (Sundberg et al., 1982; Yang and Crawley, 2009). In the 1980s the olfactory Habituation/Dishabituation test was first assessed in gerbils by (Gregg and Thiessen, 1981) and later the test has also been tried out in other species (dairy cows: Rørvang et al., 2017b, pigs: Kouwenberg et al., 2009; Aviles-Rosa et al., 2020, mice: Arbuckle et al., 2015). Yet it has only been tried out once in equids (Hothersall et al., 2010). In the study by Hothersall et al. (2010) the odors tested were of social origin (urine, feces and body odor from rubbing blankets on the fur). Despite the more obvious biological relevance of social odors, other types of odors might also be relevant to horses. These include several complex odors from, e.g., herbs and grasses (relevant in a food choice context), potentially certain soil types (as these might contain minerals horses need), and smoke and predators (as horses need to learn which odors to avoid).

This study aimed to investigate horses’ odor exploration behavior and olfactory sensitivity when exposed to non-social odors. The study aimed to make further adaptations of the Habituation/Dishabituation paradigm (Yang and Crawley, 2009) to ensure applicability and relevance to horses (adapted from Hothersall et al., 2010 and Rørvang et al., 2017b), and investigate if age, sex and gestational stage affected the odor exploratory behavior and olfactory interest of horses. Since this study was the first to test horses’ abilities to detect non-social odors and potential effects of age, sex and gestational stage, hypotheses could not be made prior to the study. Instead, the study was a hypothesis generating investigation for future research on horse olfaction and non-social odors.

## Materials and method

### Ethical considerations

The owner of the horses was informed and agreed to all experimental procedures, data collection and publication before the experiment started. All procedures were conducted in accordance with national legislation on animal experimentation by the Danish Ministry of Justice, Act. no. nr. 253 (8 March 2013) and §12 in Act. no. 1459 (17 December 2013), and met the ARRIVE guidelines (Kilkenny et al., 2010) and the ethical guidelines proposed by the Ethical Committee of the ISAE (International Society of Applied Ethology) (Olsson et al., 2017).

As the experiment was conducted during the COVID-19 pandemic, measures were taken to comply with the current precautions during the period March – April 2021. The experiment was conducted in Denmark, and hence complied with the Danish COVID-19 regulations (Sundhedsministeriet, 2021). The experiment was conducted in a separate building, distanced from other daily activities related to training, and management of horses who were not part of the experiment.

### Animals, housing and management

Thirty-five privately owned Icelandic horses aged 6 months - 25 years old (mean ± sd = 10.4 ± 8.1) participated in the tests. Three geldings, seven stallions and 25 mares were included, and the uneven sex distribution was only caused by availability of horses at the farm. The horses were kept in individual pens with sawdust bedding during nighttime and on days of testing. Horses were pastured during daytime when not participating in the tests. The horses were either native to the stud or had been kept at the stud for minimum 6 months prior to the study. All horses were handled and trained by the same trainer at the time of the study. Feed (concentrates) and fresh hay (approx. 1.5% of total body weight = 5 kg hay per day) were provided twice daily (at 0700 and 1800 h). The horses were tested in a familiar individual pen to avoid stress due to being moved, and horses were never socially isolated (minimum 4 horses (maximum 6 horses) were present in the barn during testing). The specific barn section (hereafter called “stable”), in which the testing took place, was separate from the other farm buildings and contained eight individual pens (four on each side of the rectangle stable, separated by a stable aisle). The pen sides were made of solid wood (bottom half) and metal bars (top half) and allowed the horses physical contact with neighboring horses, and visual contact with neighboring and adjacent horses (i.e., horses could see all other pens). The front side of the pen had a lower wooden part allowing the horse access to the stable aisle.

### Habituation procedure

**FIGURE 1:**
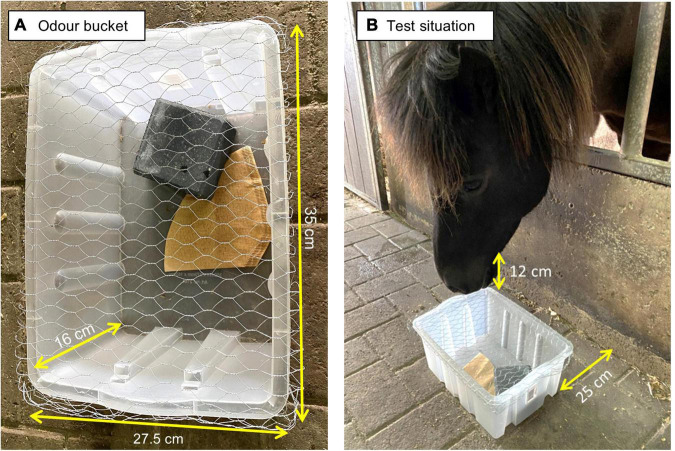
**(A)** Illustration of an odor bucket with wire mesh lid, ballast rock and filter paper (with odor sample). The odor bucket measured 35 × 16 × 27.5 cm. **(B)** Illustration of the test situation. The odor bucket was placed on the aisle floor 25 cm from the grating to the horse’s pen. Sniffing duration was measured as from when the horse’s muzzle was within the length of one horse muzzle (12 cm) away from the bucket.

Prior to testing, a habituation procedure was carried out to familiarize the horses to the equipment used, and the presence of the human experimenter. One specific odor bucket (see section “Odors buckets” below, Figure 1A) was used as a habituation bucket, which never had any odor added. The habituation procedure consisted of placing the habituation bucket in the stable aisle, in front of the horse’s home pen, 25 cm away from the metal bars (Figure 1B) allowing the horse to investigate the bucket. The horse was free to touch, sniff, lick and bite the bucket for 3 min. In order to meet the habituation criterion, the horse should approach the bucket, at least once, within the 3 min period and investigate it for more than 10 s but no more than 2 min. In addition, the horses should not display any behavior indicative of fear (such as immobilization or freezing behavior, flight responses, backwards movements or vigilant behavior (i.e., head raised above shoulder height while ears pointing forwards) neither during investigation of the bucket nor when the human experimenter approached the pen (i.e., during placement/removal of the habituation bucket). The horses had up to three 3-minute trials distributed over the course of the day (maximum 3 trials per day) in order to meet the habituation criterion (all but one horse met the criterion during these three trials). When the horse met the criterion it was ready for testing and was tested either in the afternoon of the same day (if the criterion was met during the morning trials) or on the next day.

### Odor buckets

The odor buckets (Figure 1A) were five white plastic boxes (height x length x width: 16 cm x 34 cm x 23.5 cm, 13 L; model 9950, Jem & Fix, Hørsholm, Denmark), which were covered with a wire mesh (galvanized wire (0.9 mm Ø) mesh (width: 6 mm) model 6321, Rancho, Odense, Denmark)). The wire mesh lid made the odor buckets permeable to air, and hence odors, but prevented the horses from eating, licking and touching the odor samples. The odor buckets each had a ballast weight in the shape of a concrete block (model SF-Klostersten Camfered, Type 2, pure and clean concrete, height x width x thickness: 14 cm x 21 cm x 5.5 cm, weight: 3.53 kg; IBF, Ikast, Denmark) in order to prevent the horses from tilting and/or tipping the odor buckets. All odor buckets, wire mesh and ballast stones were purchased 2 weeks prior to experimental start and were placed in the stable (Figure 1A) one week before the experiment commenced to ensure that the equipment in itself smelled familiar and similar to the horses’ home environment.

### Odor samples

Substances chosen as test odors were odor oils approved for human use: Orange oil (Citrus sinensis), peppermint oil (Mentha piperita), cedarwood oil (Cedrus) and lavender oil (Lavandula angustifolia) (Urtegaarden ApS, Allingaabro, Denmark). These substances are all complex odors (i.e., each composed of many different odorants) which were chosen as they are natural, and non-toxic odors, cheap, accessible and easy to standardize (using the build-in drop mechanism in the odor bottle). The specific four odors were chosen from 36 possible odors as we hypothesized that orange, peppermint, cedar wood and lavender would be novel to the horses, since none of these substances were found in their feed, hay or as ingredients in any products used on the farm (e.g., crèmes or soap). Moreover, we hypothesized, based on human perception, that these four odors would be perceived as different to each other. Fresh odor samples were prepared before each testing trial (approximately twice a week). Odor samples were prepared in a separate, closed room at one end of the stable. The entrance to the odor preparation room was outdoors (no direct access from the stable), hence the room was out of sight to the horses and odor contamination was limited. Odor samples were made by placing one filter paper (unbleached, light brown filter paper model 7,607, Harald Nyborg, Viborg, Denmark) in the odor bucket, and adding 10 drops (∼0.649 mL) of the odor oil to it at room temperature (Figure 1A), using the build-in drop applicator on the odor oil bottle. The filter paper absorbed and dispersed the odor oil and ensured that all odor samples had similar coloring. A separate odor bucket and corresponding wire mesh lid and ballast stone were used for each different odor in order to prevent any cross-contamination of odors. At the end of a test day, all odor buckets had the filter papers removed, and all equipment used (odor bucket, wire mesh lid and ballast weight) were cleaned with water and odorless soap. When cleaned, the materials were left to dry for at least 24 h before the next test was initiated. Odor preparation and cleaning of equipment were done in a room separate from the testing area, which were ventilated 24 h a day by two fully open windows at either end of the room. The person handling odor samples wore latex gloves for all preparation procedures.

### Developing the Habituation/Dishabituation test

Using the methods reported in Rørvang et al. (2017b) and Hothersall et al. (2010), we developed a Habituation/Dishabituation test, which investigated if horses were able to detect and distinguish between odors of non-social origin. In order to assess horses’ interest in the odors, each first presentation of an odor was used to indicate the horse’s immediate interest in the odor (as proposed in Rørvang et al., 2017b). In olfactory preference testing (e.g., as proposed by Witt et al., 2009) water is used as the odorless neutral reference point but as in Rørvang et al. (2017b) pilot testing of horses (*n* = 3 Icelandic horses, who were not part of the actual study) indicated that sniffing water resulted in horses being unwilling (or unmotivated) to approach and engage in further sniffing. To avoid this, we excluded the water sample and instead used each first presentation as indicator of horses’ immediate interest. Interest in an odor was thus measured as time spent sniffing the odor (as in the rodent version of this test, Coronas-Samano et al., 2016) at the first presentation of the particular odor, including when the horse was in direct contact with the odor bucket (Rørvang et al., 2017b).

### Test procedure

To limit potential odor contamination of the stable, a maximum of six horses were tested on one test day, and the stable door and windows were kept open. Prior to testing, a balanced odor order presentation order was made (Table 1), to ensure all possible odor presentation orders were tested. Each horse was assigned to a distinct odor order randomly when the experimenter arrived at the horse stud. In the test situation, the particular odor bucket was moved from the preparation room to the stable and placed in front of the horse’s individual pen, in the same manner as during habituation (Figure 1B). The same experimenter prepared the odor samples, and performed the tests for all horses throughout the experiment. The experimenter was not naïve to the odors used or the odor presentation order (as the person could smell the odors used), but naïve to the horses’ age, sex and gestational stage (which was provided by the horse owner after each trial). Each odor was presented three times in a row for a duration of 1 min each, with an inter-trial break of 2 min. After removal of the first odor, the horse again had a 2 min break without odor (Wesson et al., 2008) before being presented with the next odor. During all breaks, the experimenter removed the odor bucket from the stable to the preparation room to limit inter-trial contamination. After placing an odor bucket, the experimenter would move 1 m away from the horse, while still being positioned in the stable aisle directly in front of the horse being tested, in a squat position as during habituation (i.e., all horses were habituated to the experimenter).

**TABLE 1:** Odor presentation order with 1st, 2nd, 3rd and 4th odor O = orange, P = peppermint, C = cedar wood, L = lavender.

1st			2nd			3rd			4st			n
O1	O2	O3	P1	P2	P3	L1	L2	L3	C1	C2	C3	2
						C1	C2	C3	L1	L2	L3	1
			C1	C2	C3	L1	L2	L3	P1	P2	P3	2
						P1	P2	P3	L1	L2	L3	1
			L1	L2	L3	P1	P2	P3	C1	C2	C3	2
						C1	C2	C3	P1	P2	P3	1
P1	P2	P3	O1	O2	O3	C1	C2	C3	L1	L2	L3	2
						L1	L2	L3	C1	C2	C3	1
			C1	C2	C3	O1	O2	O3	L1	L2	L3	2
						L1	L2	L3	O1	O2	O3	2
			L1	L2	L3	O1	O2	O3	C1	C2	C3	1
						C1	C2	C3	O1	O2	O3	1
L1	L2	L3	P1	P2	P3	O1	O2	O3	C1	C2	C3	2
						C1	C2	C3	O1	O2	O3	1
			O1	O2	O3	P1	P2	P3	C1	C2	C3	2
						C1	C2	C3	P1	P2	P3	1
			C1	C2	C3	O1	O2	O3	P1	P2	P3	1
						P1	P2	P3	O1	O2	O3	2
C1	C2	C3	P1	P2	P3	O1	O2	O3	L1	L2	L3	1
						L1	L2	L3	O1	O2	O3	1
			O1	O2	O3	P1	P2	P3	L1	L2	L3	2
						L1	L2	L3	P1	P2	P3	2
			L1	L2	L3	O1	O2	O3	P1	P2	P3	2
						P1	P2	P3	O1	O2	O3	1

Each odor was presented 3 times, represented as 1-3 in the table. Each presentation order sample size represented as n in the last column.

### Behavioral observations

Two stopwatches (model 38.2016, TFA Dostmann GmbH & Co., KG, Wertheim, Germany) were used, one to continuously record sniffing behavior during an odor presentation, and another to time the duration of each odor presentation trial (1 min) and inter-trials pause (2 min). Sniffing was defined as the horse’s muzzle being in close proximity of (i.e., less than the length of a horse muzzle (12 cm); Figure 1B) or in direct contact with the odor bucket. Sniffing behavior was thus visually monitored and continuously recorded by direct observation (Bateson and Martin, 2021). This was done by an experienced observer naïve to the horses’ age, sex and pregnancy status, but not naïve to the specific odor being tested. Licking and biting when in contact with the odor bucket (i.e., while sniffing) as well as flehmen, backing and snorting during the odor presentation (Table 2) were recorded separately but alongside the recording of sniffing behavior by the same observer using one-zero sampling (Bateson and Martin, 2021). Olfactory exploration behavior has in humans been linked to olfactory interest (Han et al., 2022), and thus duration of sniffing and occurrence of licking and biting was used as an indicator of horses’ interest in the odors. Habituation to an odor was defined as a significant decrease in sniffing duration per presentation, measured over the three consecutive presentations of the same odor. Dishabituation was defined by reinstatement of sniffing when a new odor sample was presented.

**TABLE 2:** Ethogram of behaviors recorded during testing.

Behavior	Description
Licking	Muzzle of the horse is in direct contact with the odor bucket, or less than the length of a muzzle away from the odor bucket, with tongue protruding and touching the odor bucket at least once.
Biting	Muzzle of the horse in direct contact with the odor bucket, with open mouth and teeth touching the bucket at least once.
Flehmen*^a^*	The horse curls the upper lip backwards and inhales simultaneously in both mouth and nose. Head may be elevated and neck may be extended.
Backing	The horse takes at least two steps backwards.
Snorting*^a^*	Short powerful exhalation(s) from the nostrils

*^a^*Adapted from Christensen et al., 2005.

### Statistical analysis

The data comprised 12 repeated measures for each experimental animal; 3 tests per odor, i.e., first, second and third presentation, of 4 odors in total (Table 1). All analyses were performed using software R version 3.6.0 (2019-04-26, “Planting of a tree”) and all *P*-values were evaluated using a significance level of 5%.

#### Habituation/Dishabituation, interest and effect of age, and sex

A linear mixed-effect model was fitted to the data using R-package lme4 (Bates et al., 2015) to investigate sniffing duration. Sniffing duration data was left-skewed due to a large number of zeros and was thus log-transformed before the analysis was performed. The full model included fixed effects of trial (categorical variable with three levels: 1, 2, 3), odors (categorical variable with four levels: orange, peppermint, lavender and cedar wood), sex (categorical variable with two levels: female, male), and age (numerical variable: mean ± SD: 11.1 ± 7.4 years), and random effect of horse ID (1-36) to account for repeated measures on each horse (i.e., 12 odor exposures per horse). The model fitting showed that sex had no effect on sniffing duration, and the final model thus included all above-mentioned fixed and random effects except sex. Pairwise comparisons for each fixed effect from the model were performed using contrasts in R-package emmeans (Lenth, 2021). These comparisons investigated (1) if significant habituation (reduction in sniffing duration) occurred between successive presentations of same odors (one comparison per odor per horse; *n* = 144, Table 1), (2) whether reinstatement of sniffing (dishabituation) occurred when a new odor was presented by comparing sniffing durations between successive presentations of different odors (three comparisons per horse; *n* = 108, Table 1), (3) if any of the odors elicited more sniffing than others, and (4) if sex and age affected sniffing.

To investigate if sniffing sustained over the course of the experiment (i.e., regardless of specific odor), a linear-mixed effect model was fitted to the data, including the random effect of horse, and the fixed effects of total trials (as above), and presentation (categorical variable with three levels: first, second and third presentation). Anova analysis was used to calculate statistical significance of the overall effect of presentation.

#### Behavior

Due to the low occurrence of the behaviors, data could not be assumed to be normally distributed (evaluated in histograms), and hence non-parametric statistics were used when analyzing these count data (Siegel and Castellan, 1988). Licking and biting was analyzed separately, whereas backing and snorting was summed to form “behavior indicative of aversiveness” due to low occurrence. A Fishers Exact test for count data was used to compare if more horses expressed licking, biting, and behavior indicative of aversiveness when presented for the four odors (orange vs. lavender, orange vs. peppermint, orange vs cedar wood, etc. for all odor combinations = 12 comparisons for each of the three behaviors). The same test was used when comparing if more females than males expressed the behavior when presented to an odor (i.e., number of males vs. females expressing; licking, biting, and behavior indicative of aversiveness (snorting and backing), for orange, peppermint, lavender and cedar wood, respectively).

#### Effects of pregnancy

Of the 25 mares, eight were pregnant at the time of the study with (mean ± SD) 75 ± 31 days to expected birth when tested. To investigate whether pregnancy affected interest in the odors, a separate data set was made including only data from females. A linear mixed-effect model was fitted to the data, including fixed effects of trial and odors as above, and of pregnancy (categorical variable with two levels: yes (*n* = 8)/no (*n* = 17)). The random effect of horse was also included as above. Pairwise comparison for the fixed effect of pregnancy was performed using contrasts in R-package emmeans (Lenth, 2021), to compare pregnant females (*n* = 8) to non-pregnant females (*n* = 17). Due to the lower n in this data set, licking and biting was summed to form “appetitive behavior” and backing and snorting was summed to form “behavior indicative of aversiveness”. A Fisher exact test for count data was used to test if more pregnant than non-pregnant females expressed appetitive behavior and behavior indicative of aversiveness, separately.

## Results

One horse showed strong neophobic reactions to the odor bucket and was excluded from the tests for welfare reasons (the one horse, who did not meet the habituation criterion, see section “Habituation procedure” above). Five other horses showed no interest in the test situation (i.e., did not approach the odor bucket) when presented with lavender for the first time (*n* = 2) or when presented with lavender for the second time (*n* = 1), or when presented with cedar wood the first time (*n* = 1), or when presented with cedar wood for the second time (*n* = 1).

### Habituation/Dishabituation, interest and effect of age and sex

**FIGURE 2:**
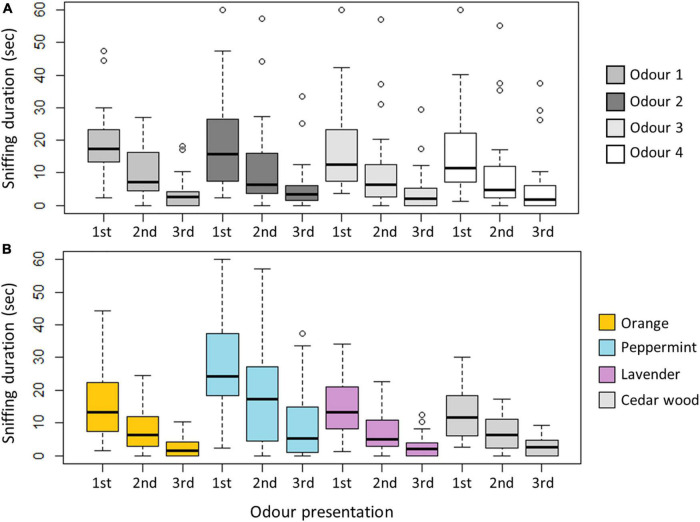
**(A)** Illustration of sniffing duration per horse during odor presentations (1st, 2nd, 3rd) over time regardless of specific odor. **(B)** Illustration of sniffing duration per horse during odor presentations (1st, 2nd, and 3rd) for all odors, orange representing orange, light blue representing peppermint, purple representing lavender and light gray representing cedar wood. For both **(A,B)** the boxes represent the 25, and 75% quartiles, the thick line inside the box represent the median and the dashed lines illustrate the range.

All horses sniffed the same odor significantly less when presented with it the second and third time (Figure 2B, Anova: F_*df*_ = 98.82_364_, *P* < 0.001). The pairwise comparisons moreover showed that this was the case for all odors (all pairwise comparisons: 1*^st^* vs 2*^nd^*: estimate ± se: 7.71 ± 0.97, df = 364, t-ratio = 7.04, *P* < 0.001 and 2*^nd^* vs 3*^rd^*: estimate ± se: 5.90 ± 0.97, df = 364, t-ratio = 6.06, *P* < 0.001).

When presented with a new odor after the third odor presentation, sniffing duration increased significantly (1*^st^* vs 3*^rd^*: estimate ± se: 13.61, df = 364, t-ratio = 14.01, *P* < 0.001), in all but 3 cases (Table 3 and Figure 2A).

**TABLE 3:** Results from final model, the pair wise comparisons of each pair of 3rd and 1st odor presentations, with sample size, estimate ± se, df, *t*-ratio and *P* listed.

Third vs first odor presentations	N	Pairwise comparison Estimate ± se,	Df	*t*-ratio	*P*
Orange 3rd vs. peppermint 1st	9	−24.63 ± 1.47	364	−16.78	<0.001
Orange 3rd vs. lavender 1st	8	−13.03 ± 1.46	365	−8.77	<0.001
Orange 3rd vs. cedar wood 1st	9	−12.38 ± 1.49	365	−8.34	<0.001
Peppermint 3rd vs. orange 1st	9	−2.58 ± 1.47	364	−1.76	0.84
Peppermint 3rd vs. lavender 1st	9	−2.01 ± 1.49	365	−1.35	0.97
Peppermint 3rd vs. cedar wood 1st	8	−1.36 ± 1.49	365	−0.92	1.00
Lavender 3rd vs. orange 1st	9	−14.19 ± 1.49	365	−9.51	<0.001
Lavender 3rd vs. peppermint 1st	9	−25.21 ± 1.49	365	−16.90	<0.001
Lavender 3rd vs. cedar wood 1st	9	−12.97 ± 1.51	367	−8.59	<0.001
Cedar wood 3rd vs. orange 1st	9	−14.83 ± 1.49	365	−9.94	<0.001
Cedar wood 3rd vs. peppermint 1st	9	−25.85 ± 1.49	365	−17.33	<0.001
Cedar wood 3rd vs. lavender 1st	8	−13.61 ± 0.97	364	−14.01	<0.001

During the course of the experiment, sniffing duration of the four odors did not vary between presentations regardless of odor (Figure 2A, Anova: F*_df_* = 0.50_9_, P = 0.87), indicating that the horses’ interest in the test situation/the odors persisted over time (total test duration per horse: 20 min).

**FIGURE 3:**
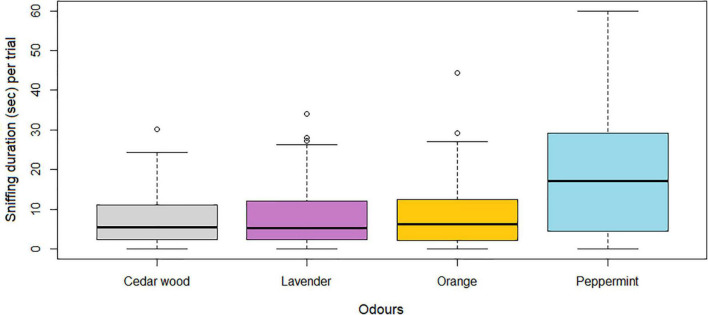
Illustration of the total sniffing duration (seconds) of each odor per horse. Orange represent orange, light blue represents peppermint, purple represents lavender and light gray represents cedar wood. The boxes represent the 25, and 75% quartiles, the thick line inside the box represent the median and the dashed lines illustrate the range.

Sniffing duration was significantly affected by the specific odor (Anova: F_*df*_ = 55.11_366_, *P* < 0.001), and greatest when horses were exposed to peppermint compared with all other odors (Figure 3).

The model fitting showed that sex had no effect on sniffing (Anova: F_*df*_ = 0.19_31_, *P* = 0.66), but although only a tendency, age effected sniffing and was kept in the model (Anova: F_*df*_ = 2.77_32_, *P* = 0.10). A *post hoc* analysis using the same final model including the interaction of odor and age showed that the effect of age was specifically linked to sniffing of cedar wood, with younger horses sniffing significantly longer (estimate ± se: −0.31 ± 0.14, df = 86, *t*-ratio = −2.23, *P* = 0.030). Seven of the 0-5 year old horses sniffed cedar wood for more than 30 seconds at first presentation. In addition, there was a tendency for younger horses to sniff peppermint less than older horses (estimate ± se: 0.28 ± 0.14, df = 362, *t*-ratio = 2.01, *P* = 0.046), with horses younger than 5 years (*n* = 6).

### Behavior

**FIGURE 4:**
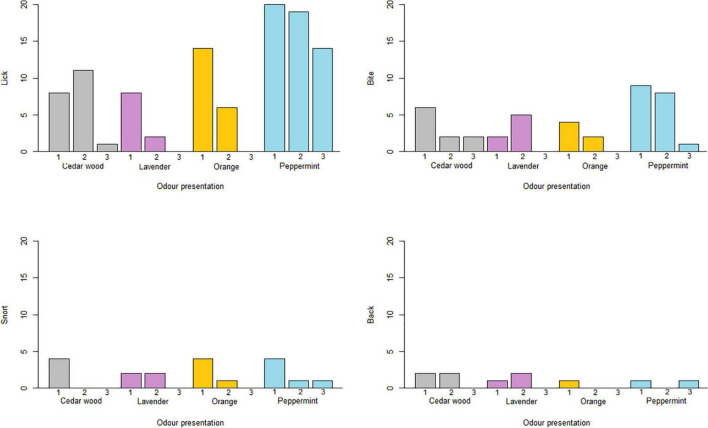
The total number of horses expressing licking, biting, snorting and backing separately when presented with each of the odors 1st, 2nd, and 3rd, time. Orange represent orange, light blue represents peppermint, purple represents lavender and light gray represents cedar wood.

Licking was the most common behavior, which was expressed by all horses, but not by all horses for all odors (Figure 4). Biting was the second most common although less frequent than licking. Snorting and backing were generally rare and restricted to only 11 horses, and thus these two were grouped to form “behavior indicative of aversiveness”. Flehmen was not observed at all. More horses showed licking behavior when presented with peppermint, than lavender or cedar wood (Fishers Exact test: peppermint vs. lavender & peppermint vs. cedar wood: OR = 4.40, *P* = 0.0068), but there were no other differences between other pair comparisons. Generally, fewer horses expressed biting (Figure 4), but significantly more horses bit the peppermint sample, compared to the lavender sample (Fishers Exact test: OR = 0.18, *P* = 0.045), whereas no other pair comparisons were significantly different (Fishers Exact test: all *P*-values > 0.050). Due to the low occurrence of behavior indicative of aversiveness (snorting and backing summed) per odor, statistical analyses was not possible, but this behavior was not connected to any particular odors and did not increase or decrease over time (Figure 4). Lastly, equally many females and males expressed licking, biting, and behavior indicative of aversiveness (Fishers Exact tests: all *P*-values > 0.10).

### Effect of pregnancy

**FIGURE 5:**
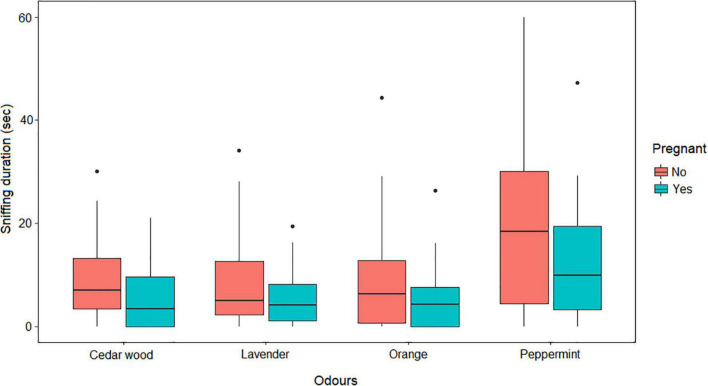
Illustration of sniffing duration (seconds) per odor (orange, peppermint, lavender and cedar wood), divided by pregnant (blue) and non-pregnant (red) females. The boxes represent the 25, and 75% quartiles, the thick line inside the box represent the median and the vertical error bars illustrate the range.

The model fitting on the data set including only females, showed that pregnancy had a significant effect on sniffing duration (Anova: F_*df*_ = 5_23_, *P* = 0.030), with pregnant females sniffing the odors less than non-pregnant females (Figure 5). Although sniffing duration was affected by pregnancy, equally many pregnant and non-pregnant mares expressed behavior indicative of aversiveness (Fisher’s Exact test: *P* > 0.050) but there was a tendency for fewer pregnant females expressing appetitive behavior (Fisher’s Exact test: *P* = 0.089).

## Discussion

In this study, horses were able to detect and distinguish between four complex odors; orange, peppermint, lavender and cedar wood. The Habituation/Dishabituation test paradigm developed for the horses worked well to test olfactory abilities of horses. Over the course of the experiment, the overall sniffing duration did not change significantly regardless of odor (Figure 2A), hence horses’ interest in the testing situation (and the odors) persisted during the course of the experiment. Horses showed increased interest (significantly longer sniffing duration) when presented to peppermint compared with all other odors. More horses expressed licking behavior when presented to peppermint compared to cedar wood and lavender, and more horses exhibited biting behavior when presented to peppermint compared to lavender. Young horses sniffed cedar wood for longer than older horses (and peppermint less, tendency), and behaviors indicative of averseness (i.e., snorting and backing) was generally low, and did not increase over time (regardless of odor). In addition, pregnant mares sniffed all odors less than non-pregnant mares, and less pregnant mares expressed appetitive behavior (licking and biting) when exposed to the odors (tendency).

In 2010, Hothersall et al. were the first to try adapting the Habituation/Dishabituation test for horses using social odors (i.e., urine, feces or fleece fabric previously rubbed on the fur of a companion), but the current study is the first to test horses on complex odors with no social reference to the horse. The horses readily habituated to the odors when presented successively, and sniffed new odors significantly longer, hence both habituating and dishabituating to the odors according to the definition of the terms (see section “Developing the Habituation/Dishabituation test”). Compared to the test situation made by Hothersall et al. (2010), this study made some further adaptations of the test to improve the validity of the results: To avoid any human cueing and other effects from humans physically presenting/holding the samples, the odor boxes in the current experiment were presented to the horses on the stable aisle. The human experimenter only brought and retrieved the samples. The wire mesh on top of the odor buckets further ensured that horses were unable to touch the samples to avoid the mixing of smell and taste. In previous studies of olfaction in animals, it has been common to add a presumably neutral odor control (usually water) (e.g., cattle (Rørvang et al., 2017b), mice (Cho et al., 2018), rats (Tarland and Brosda, 2018)). In the current study, however, the water control was excluded to avoid horses losing interest in sniffing something odorless, which has previously been proven a challenge when testing cattle (Rørvang et al., 2017b). Since the test paradigm relies on the animal voluntarily investigating the odors, it is essential that appetitive activity is sustained throughout the test. The overall sniffing duration for odor samples presented the 1st, 2nd, 3rd, and 4th time (i.e., regardless of specific odor) did not decrease or increase in the current experiment, hence motivation to investigate sustained over the course of the experiment. It should be noted, that although odor samples were prepared in the same manner and with same volume of odor oil, the vapor pressure of each odor could have differed resulting in varying odor received per sniff. However, only two horses failed to approach the odors (one on lavender and one on cedar wood) after a first presentation, suggesting that the majority of horses were motivated to investigate the odors regardless of specific odor, time, or order of odor presentation. Behaviors indicative of aversiveness were infrequent (mean (range) of occurrence per odor presentation: snorting: 1.6 (0 - 4), backing: 0.8 (0 - 2)), and horses expressing these did not continue to do so, indicating these behaviors might have been a result of novelty rather than averseness.

As previously done with cattle (Rørvang et al., 2017b), our horses were tested in a social setting. This is the greatest change from the original rodent tests, which are done in an enclosed cage with only the focal animal present, e.g., Tarland and Brosda (2018). This setting was chosen to limit the negative effects of social isolation. Horses were tested from a familiar individual pen, and had companions nearby, although not in the neighboring pens to limit odor contamination. The disadvantage to this setting is a potential risk of social transmission of fear or induced curiosity caused by reduced fear (Rørvang et al., 2015). The effect of the presence of conspecifics may however be more profound when the situation is more frightening, making social transmission of fear less likely in this particular situation as all animals were habituated to the test situation beforehand. Occurrence of behavior related to fear or aversiveness (i.e., vigilance, snorting, backing and flight) were uncommon during habituation and later during the test. During the test, behavior indicative of aversiveness might have been an indication of activation of the trigeminal nerve (Aleman et al., 2013). The olfactory and trigeminal systems have a close relationship, and some odors can trigger the trigeminal nerve (Frasnelli et al., 2007). In the current study, it was not possible to elucidate if the behavior indicative of aversiveness observed was a result of a stimulation of the olfactory nerve, the trigeminal nerve, a learned response to the olfactory stimuli or a combination of these. Since the odors were novel to the horses, and as the horses expressing behavior indicative of aversiveness in a first presentation did not continue to do so in subsequent presentations of the same odor, it is likely that this behavior was caused by novelty. Horses often react with fear-related behavior to novelty (Visser et al., 2003; Lansade et al., 2007), and presentation of an unknown odor for the first time, is likely to elicit such behavior.

Of the four odors presented, peppermint evoked the most investigation (longest sniffing duration) for all horses. Peppermint has also previously been found to increase activity in other species, e.g., captive mice (Umezu et al., 2001), dogs (Graham et al., 2005), and zoo-kept lions (Powell and Powell, 1995). In the lion study, peppermint also stimulated more species-specific behavior (back rolling). All types of mints, including peppermint, are among the oldest herbs used for medical purposes (Balakrishnan, 2015) and is botanically related to catnip (Ellis and Wells, 2010). Catnip is known to both encourage play behavior in cats, but can also increase sleep and hence reduce activity (Ellis and Wells, 2010). From human studies, variants of mints (eucalyptus) has also been rated as one of the more pleasant odorants like strawberry and shampoo as opposed to the smell of dirty socks and sweat (Fredenzi et al., 2013). Peppermint is commonly used as flavor in horse treats and feedstuffs (e.g., Team, 2015) as well as in many types of fluent electrolyte mixtures or insect repellents (e.g., Cointreau, 2014). Horses may thus have had an already established association with peppermint. This theory was further supported by the high number of horses expressing licking and biting behavior when sniffing the peppermint samples (20 out of 35 horses at the first presentation of peppermint). Horses may thus have perceived peppermint odor as edible, and hence expressed more behavior linked to eating. Following communications with the horses’ owner/trainer, it was nonetheless noted that none of the horses had been fed any treats or feed with peppermint, eucalyptus or other mint flavors for at least the period they were in this particular stable (minimum 6 months). The latter moreover means that none of the 0-5 years old horses tested (*n* = 14) had ever been exposed to peppermint (or other mints), since they grew up at this stable, and were never handled by other trainers. This adds further support to a theory suggesting peppermint to evoke an innate interest in horses. Peppermint may thus, in addition to activating the olfactory nerve, have activated the facial and glossopharyngeal nerve (taste innervation of the tongue), resulting in the licking and biting behavior. For the young group of horses naïve to mints, this could indicate that these may have been able to link smell with taste (as, e.g., humans, Schifferstein et al., 2020), which has never been demonstrated before (Rørvang et al., 2020).

This study is the first to illustrate an effect of age on olfactory interest in horses. Research on cedar wood is not abundant, but there is some evidence for a relaxing effect of inhaling the odor in rats (Kagawa et al., 2003). This effect could be speculated to affect horses’ inhalation behavior, resulting in less sniffing in some horses. The age effect on sniffing of cedar wood odor could be a result of a deterioration of the older horses’ (mean age of older horses: 16) olfactory abilities making them express less odor exploration behavior. In human studies, patients with reduced olfactory function has been shown to express less frequent odor exploration behaviors, compared with patients with normal olfactory function (Han et al., 2022). However, another possible explanation for less sniffing of cedar wood in older horses, could be prior exposure to similar cedar-like odors such as pine trees or wood materials used in fencing or stable inventory. Pinewood is abundant in Denmark, and it is thus possible that some horses (especially older horses) already had been exposed to this odor lowering their interest in the odor. In contrast to our finding, Hothersall et al. (2010) found no effect of age on investigation of social odors (mares and foals were tested). The underlying reason for the different results could be the social nature of the odors used by Hothersall et al. (2010) and the complex, non-social odors used in this study. Due to social odors likely playing a role in sexual behavior, age differences might be more pronounced and biologically relevant in this context compared with non-social odors (Pluháèek et al., 2019). The response found in the young horses are potentially more ‘pure’ as individuals are affected by their environment throughout their lives, and hence older horses may learn to associate certain situations and emotions with an odor (Salesse and Dormont, 2017). As a result, horses may come to “like” or “dislike” odors, which they did not innately have any association to. More studies are needed to confirm if olfactory abilities of the horse decline with age, and to outline if and how this affects the handling of horses.

This study is the first to report an effect of pregnancy on olfactory interest in mares, which is somewhat in accordance with other findings on dairy cattle (Rørvang et al., 2017b; Jensen and Rørvang, 2018; Rørvang, 2018). However, dairy cow olfactory responsiveness (or preference) have only been shown to change with regard to social odors (amniotic fluids), and hence cows may, like horses, differ with regards to complex, non-social odors. Outside parturition, many ungulates are repulsed by odors linked to the placenta and/or the amniotic fluids (e.g., golden hamster: Richards, 1966, gerbils: Elwood, 1977, Sheep: Levy et al., 1983). As parturition approaches, however, the female becomes increasingly responsive toward cues from the young, and some of these cues are of olfactory nature (Levy and Nowak, 2017). We speculate that although some odors may have little or nothing to do with the young, the hormonal change in the female might affect her response to both social odors and non-social odors (i.e. like a side-effect). The effect of pregnancy was, in this case, not linked to any odors in particular, but numerically the difference was greatest within peppermint, orange and lavender. Lavender is, like cedar wood, an odorant associated with anxiolytic effects (Schuwald et al., 2013). For instance, shelter dogs exposed to lavender have been found to reduce activity and vocalizations and in turn spend more time resting (Graham et al., 2005), and travel-induced excitement could be lowered in dogs during transit when exposed to lavender (Wells, 2006). Travel sickness in pigs was also alleviated when pigs had access to straw sprayed with lavender (Bradshaw et al., 1998), and dressage horses exposed to lavender aromatherapy have lower heart rate variability leading the authors to conclude that lavender have an immediate calming effect on horses (Baldwin and Chea, 2018). It was therefore specifically surprising that pregnant mares indicated some level of avoidance of lavender, unless it could have potential harmful effects on the fetus. Future studies should therefore focus on testing olfactory interest of mares both pre- and post-partum in order to fully understand how olfactory interest changes with gestational state. This is especially important in relation to aromatherapy as some odors may be more or less suitable for various groups of horses. A large amount of work also remains with testing the already available odorous remedies (i.e., aromatherapies, pheromones, Draaisma, 2021) on the market to establish if these have a real purpose of use.

In conclusion, this study adds important information to the basic knowledge and understanding of equine olfaction. Horses were able to detect and distinguish between orange, peppermint, lavender and cedar wood, and peppermint elicited most interest and appetitive behavior. Pregnancy reduced sniffing of all odors, and age affected sniffing of cedar wood. The results can aid the understanding of horses’ behavioral reactions to different odors, and in the future, it may be possible to relate these to the physiology and health of horses. Odors may constitute a source of environmental enrichment for horses either directly as pleasant scents, or secondary as new scents to already existing enrichment materials.

## Data availability statement

The raw data supporting the conclusions of this article will be made available by the authors, without undue reservation.

## Ethics statement

Ethical review and approval was not required for the animal study because the owner of the horses was informed and agreed to all experimental procedures, data collection and publication before the experiment started. All procedures were conducted in accordance with national legislation on animal experimentation by the Danish Ministry of Justice, Act. no. nr. 253 (8 March 2013) and §12 in Act. no. 1459 (17 December 2013), and met the ARRIVE guidelines (Kilkenny et al., 2010) and the ethical guidelines proposed by the Ethical Committee of the ISAE (International Society of Applied Ethology) (Olsson et al., 2017). Written informed consent was obtained from the owners for the participation of their animals in this study.

## Author contributions

MR applied for, and was later awarded the funding for the study. MR designed the Habituation/Dishabituation test and performed the experimental work with virtual participation from JY and KN. MR was in charge of data processing and statistical analyses. KN and JY participated in the discussion of statistical procedures and subsequent results. KN and MR collaborated in writing the first draft of the article, and all authors contributed in discussion, proofreading, and fine-tuning the final draft for publication. All authors contributed to the article and approved the submitted version.
